# Capital Formation, Green Innovation, Renewable Energy Consumption and Environmental Quality: Do Environmental Regulations Matter?

**DOI:** 10.3390/ijerph192013562

**Published:** 2022-10-19

**Authors:** Xueying Meng, Tianqing Li, Mahmood Ahmad, Guitao Qiao, Yang Bai

**Affiliations:** Business School, Shandong University of Technology, Zibo 255000, China

**Keywords:** capital formation, environmental regulation, green innovation, renewable energy, environmental quality

## Abstract

The world economy continues to witness a steady rise in carbon emissions, which makes it challenging to fulfill the terms of the Paris agreement on reducing greenhouse gas emissions. In this context, countries worldwide enact environmental regulations to curtail environmental pollution to promote sustainable development. However, the importance of environmental regulations has not been fully validated in the previous literature. In addition, the concurrent roles of capital formation, green innovation, and renewability cannot be overlooked. Against this backdrop, this study selects data from G7 countries from 1994 to 2019 to explore the effect of environmental regulations, capital formation, green innovation, and renewable energy consumption on CO_2_ emissions. In order to achieve the above research objectives, we employ the Method of Moments Quantile Regression (MM-QR) for empirical analysis. The results reveal that capital formation significantly enhances environmental quality by reducing CO_2_ emissions across all quantiles (10th–90th). Environmental regulations show a significant and negative impact on CO_2_ emission mainly at the middle and higher emissions quantiles, while the effect is insignificant at lower quantiles (10th). Moreover, green innovation and renewable energy consumption mitigate CO_2_ emissions across all quantiles (10th–90th), while economic growth deteriorates environmental quality in G7 countries. The panel granger causality results indicate the unidirectional causality running from capital formation, environmental regulations, and renewable energy towards CO_2_ emissions, which implies that any policy related to these variables will Granger cause CO_2_ emissions but not the other way round. Based on the findings, important policy implications are proposed to promote sustainable development in G7 countries.

## 1. Introduction

Climate change is shaking the foundation of modern civilization, and therefore, it is jeopardizing the well-being of this planet, which accommodates more than 7 billion people. Unfortunately, no nation or region is exempt from the effects of climate change, as the world has seen a tremendous rise in environmental problems. Many recent studies have identified unsustainable consumption and production practices as key contributors to environmental degradation and climate change [1,2]. In recent years, countries have focused their attention on sustainable economic development and environmental issues. The fundamental reason for this concern is that economic activities (i.e., manufacturing and transportation) require a large amount of energy, increasing resource scarcity and rising environmental degradation. 

In response to climate change, many countries have begun to develop strategies and incentives known as environmental regulations. The implementation of environmental laws and green taxes is an effective way to stimulate the implementation of climate change policies. Ahmed et al. [3] argue that strict environmental laws and regulations for ecological sustainability can play a key role in reducing carbon to a certain extent. Excessive consumption of non-renewable energy will lead to the deterioration of environmental quality [4]. Sinha et al. [5] believed that we could only create a sustainable social environment by replacing traditional energy with renewable energy and improving energy efficiency. 

Severe resource and environmental crises are prominent problems that every country faces today. However, the key to solving this problem is to develop a green economy to carry out technological transformation and replace traditional high-carbon technology with low-carbon and zero-carbon green technology [6,7,8], as well as promote enterprise transformation by green development through innovation and change the business model that achieves economic gain at the cost of destroying the natural environment. Green technology innovation may be the only way to achieve win–win economic and ecological benefits [9]. Encouraging enterprises to carry out green innovation, develop new energy, and use the low-carbon nature of clean energy can promote economic growth and ensure environmental quality. Environmental regulations and other policy tools can stimulate the willingness of enterprises to produce green innovation and then motivate enterprises to invest capital and human resources in carrying out green innovation behavior. Green innovation can lead innovation to develop in the direction of environmental protection. It requires that the application of new technologies must be able to reduce the input of resources, reduce the damage to the environment, and ease the pressure of resource cost. Only in this way can we promote a virtuous circle of regional economic development.

Minimizing pollution from economic activities has recently been the top priority of most economies, including the G7 countries. As a result, countries worldwide are conducting extensive research for alternate and renewable energy resources and green technologies to move away from non-renewable energy sources (i.e., coal, oil, and gas). Capital formation and environmental regulation can play a pivotal role in achieving sustainable development goals (SDGs). However, research integrating these crucial factors into the same environmental policy framework is scant. In this context, it is pertinent to examine the unclear linkage between capital formation, green innovation, environmental regulations, renewable energy consumption, economic growth, and CO_2_ emissions in the context of G7 countries. In addition, the motivation of this research stems from the United Nations’ SDGs (7 and 13) to be attained by 2020, which is rarely in the current literature.

This study contributes to the environmental economics literature in the following ways. Firstly, it explores the relationship between capital formation, environmental regulation, and green innovation in the presence of renewable energy and economic growth in G7 countries. In addition to the long-run association, the causal linkage is investigated to make the right policy recommendations. As per the authors’ knowledge, this is the first study that combines these factors into the same environmental policy framework for G7 countries. Thus, the current research is distinct from previous studies in terms of the scope by considering G7 nations that have received less or no attention earlier. Secondly, this study uses the Method of Moments Quantile Regression (MM-QR) for empirical analysis. Therefore, the additional uniqueness of the current study is that it has used the latest estimation methods. Thirdly, this study provides practical policy implications for G7 countries to attain some SDGs set by the United Nations that are closely linked to energy transition (SDG7) and environmental sustainability (SDG13). 

The research framework of this paper is structured as follows: The first part is the introduction, which briefly expounds on the research background, research significance, and research content. The second part is the literature review, which summarizes the relevant research results of scholars in this field and puts forward the development trend of the current field based on it. The third part is the theoretical framework, data, and test methods, including variable selection and definition, data source, and model design. The fourth part is the results and discussion. Based on the research design in the third part, the empirical analysis is carried out to estimate the relationship among study variables, and the research results are obtained and further discussed. The fifth part is the conclusion and policy enlightenment, which summarizes the empirical analysis results, puts forward specific measures to be implemented, reviews the whole research, and points out the research shortcomings and room for improvement.

## 2. Literature Review

Capital formation is the result of investment behavior. The importance of investment structure to gross capital formation varies with its scale [10]. Based on the theory of capital formation, a good solution to the problem of capital, with natural resources, labor, and other factors of production, can achieve long-term sustainable economic development. In this perspective, capital formation can play an imperative role in achieving climate-related goals to accelerate economic growth without hurting environmental quality. However, limited studies have been devoted to examining the linkage between capital formation and environmental quality. Existing research does not reach an agreement on the association between capital formation and CO_2_ emissions. For instance, Rahman and Manzoor [11] investigated the impact of capital formation on CO_2_ emissions from 1980 to 2016 in Pakistan. They found that capital formation deteriorates the environmental quality by escalating emissions. Likewise, Gao et al. [12] also found that capital formation dampens environmental quality in China. They further added that capital formation increased CO_2_ emissions from 2436 (Mt) to 5089 (Mt) over the 2007–2017 period. A recent study by Mujtaba et al. [13] disclosed that capital formation exerts a positive impact on CO_2_ emissions in the short run, while it shows a negative but insignificant effect in the long run. Likewise, Adebayo et al. [14] reported no significant association between capital formation and CO_2_ emission in the case of Egypt. 

The innovation, promotion, and operation of green technology are inseparable from strong financial strength, and capital formation should be accelerated to meet the demand for funds for green innovation. There is a significant causal relationship between capital formation, income, and energy consumption. The energy crisis in developing countries has seriously drained its economy and posed a great threat to sustainable development. In this context, Khan et al. [15] sought an effective way to promote national economic growth and capital formation, that is, to encourage foreign investors to participate in green energy generation projects that can help to fulfill the energy requirements and, at the same time, help to lower the environmental pollution. Other scholars also propose solving the energy shortage problem in Pakistan by developing a green economy [16].

Green innovation is a spiral innovation process from “utilization” to “exploration”, and then from “exploration” to “utilization”. Its essence is to pursue the common realization of economic, social, and environmental benefits. The implementation of a green innovation strategy can effectively improve the energy utilization rate, improve environmental quality, and accomplish sustainable development by enhancing technological innovation ability and increasing the share of renewable energy in the energy mix [17]. In the context of Germany’s energy transition, the idea that technological innovation is the driving force of renewable energy has been validated [18]. In the process of exploring the internal influencing mechanism of technological innovation and green development, Cheng et al. [19] found that the spatial agglomeration effect was significant, so they believed that technological innovation advantage could promote green growth. Technological innovation needs a large amount of capital expenditure and talent introduction, and the establishment and improvement of external incentive mechanisms are indispensable. Among them, environmental regulations are of great significance for promoting green innovation in enterprises. The combination of various environmental regulation means can encourage the growth of green innovation in enterprises [20].

Hashmi and Alam [21] looked into the association between environmental regulations, technological innovation, and CO_2_ emission in the context of OECD countries. The findings from the DK error methods indicate that a 1% increase in environmental regulations decreases emissions by 0.03% and decreases CO_2_ emissions by 0.017% due to the 1% increase in green innovations. Ahmad et al. [7] examine the dynamic relationship between technological advancement and carbon footprint using the EKC hypothesis. According to their findings, technological progress can aid in slowing down environmental deterioration and confirm the existence of EKC. In another study, Ahmad et al. [22] argue that a country’s degree of development significantly impacts the linkage between innovation and environmental quality. Their findings demonstrate that eco-innovation has a considerable negative impact on environmental deterioration in G7 nations and that eco-innovation is more effective at reducing emissions in G7 economies than in developing economies. In general, green innovation can create a good social environment and improve environmental quality. These studies also provide strategic recommendations for countries to achieve economic growth without hurting environmental quality. 

Environmental problems are no longer a single problem of individuals or a certain country but a global problem. Protecting the ecological environment and improving environmental quality have become the top priority in economic development. The traditional coal industry seeks economic growth by consuming natural resources and destroying the environment, which is not a sustainable development model. Under the voice of environmental protection, it is urgent for enterprises to carry out economic transformation. Only the development and utilization of renewable energy, the development of a green economy, and the implementation of sustainable development can realize an economy’s healthy and stable development. Improving the utilization rate of renewable energy is an effective way to improve environmental quality [23]. Increasing the consumption of renewable energy to reduce CO_2_ emissions, and strengthening the cultivation of educational awareness to assist, makes contributions to shaping good environmental quality [24].

Driven by the goal of carbon peak and carbon neutrality, new energy technology innovation has become the new core competitiveness. Accelerating the pace of reducing carbon emissions has played a guiding role in green technology innovation. Methodically adjusting the industrial and energy structure, mining and developing renewable energy, and considering economic growth and green transformation are effective ways to defuse the resource and environmental crisis. The formation of fixed capital usually increases in the form of technological innovation [25], and endogenous technological innovation needs a large amount of capital input and support. At the same time, enterprises can obtain economic benefits through a green technology innovation strategy. The introduction and imitation of exogenous technology cannot become the backbone of economic development. Improving independent innovation and stimulating the internal driving force of innovation is the key factor leading to technological change and economic development so as to find the guarantee of long-term development in the current resource and environmental crisis. Green innovation can achieve not only economic benefits but also improve environmental quality by integrating renewable resources and increasing the consumption of renewable resources and, finally, realizing the green and sustainable development of a social economy.

Based on the literature review, it can be concluded that the effect of capital formation, environmental regulations, green innovation, and renewable energy consumption on environmental quality are ambiguous. Most scholars focus on the causal and logical relationship and interaction between economic growth and influencing factors of CO_2_ emissions and ignore the important aspects of capital formation and environmental regulation in the environmental policy framework. Therefore, this study evaluates how capital formation, environmental regulation, and green innovation influence CO_2_ emissions in G7 countries.

## 3. Theoretical Framework and Model Construction

### 3.1. Theoretical Framework

This paper studies the impact of capital formation, green innovation, and environmental regulations on environmental quality in G7 countries. Based on the Porter theory [26], the rise and fall of a country is based on whether it can gain advantages in international competition and whether the enterprise has innovation ability and technological advantages. Under the United Nations’ SDGs, enterprises worldwide have also learned that green innovation with a better capital structure is a key factor in developing a sustainable economy. Developing a green economy can create competitive industries in international competition and seek a road of long-term development [27]. Technology is the main driving force of economic growth, which is one of the views of macroeconomic theory. The development of a green economy is inseparable from the development of green technology. Studies have shown that green innovation can curb carbon emissions and positively contribute to alleviating environmental pressure [28].

Economic growth drives the demand for energy consumption [29], leading to environmental quality deterioration [30]. In order to curb the deterioration of the environment, countries have put forward a number of countermeasures such as carbon taxes, taxation on fossil fuel imports, etc. However, environmental legislation cannot ensure environmental sustainability unless it is effectively enforced. Existing studies have proved that improving the institutional system plays an important role in ensuring the effective implementation of environmental regulations [31]. Environmental regulations may not only reduce emissions directly but can also boost renewable energy, which contributes to enhancing environmental quality.

Based on the theoretical framework and previous studies of [13] and [32], we developed the following model

(1) $${{CO}_{2}}_{\mathrm{it}}=\alpha_{0}+\beta_{1}{CF}_{\mathrm{it}}+\beta_{2}{ER}_{\mathrm{it}}+\beta_{3}{GI}_{\mathrm{it}}+\beta_{4}{RE}_{\mathrm{it}}+\varepsilon_{\mathrm{it}}$$

In the above model, “i” represents the cross-section (G7 countries), and “t” indicates the annual time of the data (1994–2019). In addition, “*α*” means the intercept term, “*β*” indicates slope, and “*ε*” represents stochastic disturbance. CO_2_ is a measure of environmental quality calculated using CO_2_ emission (tones per person). CF stands for capital formation, measured by gross capital formation (constant 2015 USD). ER indicates environmental regulation, with environmental taxes as the method of calculation. GI is short for green innovation, measured by patents on environmental technologies (% of total). GDP represents economic growth, measured in per capita (constant 2010 USD).

### 3.2. Data

In order to study the effect of capital formation, green innovation, and environmental regulations on carbon dioxide emissions, this study focused on G7 countries (United States, Britain, Germany, France, Japan, Canada, and Italy). These seven countries are the world’s most advanced countries concerning economic growth, innovation, and renewable energy production and consumption. Given that the data on environmental regulations starting from 1994 and the data on green innovation last until 2019, the data range from 1994 to 2019 is selected. Data on real GDP per capita and gross capital formation are obtained from the World Development Index, data on renewable energy consumption and carbon dioxide emissions are obtained from Our World in Data, and environmental taxes and percentage of environmental technology patents are obtained from the OECD database.

The variable framework of the empirical analysis includes environmental quality measured by carbon dioxide indicators as the dependent variable, capital formation, environmental regulation, and green innovation as independent variables, controlling for economic growth and renewable energy consumption. The measurement standards and data sources of the above variables are shown in Table 1.

### 3.3. Estimation Methods 

#### 3.3.1. Cross-Sectional Dependence Test

First of all, in term of the estimation strategy, we performed a cross-sectional dependency test. Full dependence is often shown in the panel data, and the validity of the estimates declines seriously if ignored. Therefore, to avoid the contingency of the estimates, Pesaran’s [37] cross-sectional dependence method is adopted. The test equations for the CD are as follows:

(2) $$CD=\sqrt{\frac{2T}{N\left(N-1\right)}}\left(\sum_{i=1}^{N-1}\sum_{j=i+1}^{N}{\hat{\rho }}_{\mathrm{ij}}\right)$$

#### 3.3.2. Slope Heterogeneity Test

After determining whether cross-section dependence exists, the next step is to confirm slope heterogeneity in the panel. Due to the differences in the economic strength and the policy environment of the G7 countries, to explore whether the transmission of the proposed mechanism of influence varies between samples in different countries, this research employed the slope homogeneity method put forward by Pesaran and Yamagata [38]. Analyzing whether the empirical observations match the theoretical results by heterogeneity helps to test the correctness of the conduction mechanism proposed here. The test equations for the slope heterogeneity are as follows:

(3) $$\tilde{\Delta }SH=\sqrt{\frac{N}{2K}}\left(\frac{1}{N}\tilde{S}-k\right)$$

(4) $$\tilde{\Delta }ASH=\sqrt{\frac{N\left(T+1\right)}{2k\left(T-k-1\right)}}\left(\frac{1}{N}\tilde{S}-k\right)$$

#### 3.3.3. Unit Root Test

Before the long-run estimation methods, it is recommended to examine the variables’ stationary properties. If there is a unit root in the sequence, the variable is not stable, causing pseudo-regression in the regression analysis. To ensure the reliability of the empirical results, this paper applied the CIPS and CADF methods of unit root Pesaran [39]. The test equations for the unit root are as below:

(5) $${\Delta CA}_{i,t}=\phi_{i}+\phi_{i}Z_{i,t-1}+\phi_{i}{\bar{\mathrm{CA}}}_{t-1}+\sum_{l=0}^{P}\phi_{\mathrm{il}}\Delta {\bar{\mathrm{CA}}}_{t-1}+\sum_{l=0}^{P}\phi_{\mathrm{il}\Delta }{CA}_{i,t-1}+\mu_{\mathrm{it}}$$

#### 3.3.4. Westerlund Test for Cointegration

The role of the co-integration is to test whether the causal relationship described by the regression equation established in this paper is a pseudo-regression and to detect whether there is really an equilibrium dependence between the variables. Studying the co-integration relationship between the variables helps to study the quantitative regularities between the variables. Therefore, this paper adopted the method of Westerlund [40]. test for co-integration. This method can not only solve the problem of ignoring pseudo-regression in traditional measurement models but also overcome the weakness of establishing differential models to ignore the information on level variables. The Westerlund test is described by Equation below:

(6) $$\alpha_{i}\left(L\right)\Delta y_{\mathrm{it}}=\delta_{1i}+\delta_{2i}t+\alpha_{i}(y_{it-1}-{\beta^{\prime }}_{i}x_{it-1})+\lambda_{i}{\left(L\right)}^{\prime }v_{it}+e_{it}$$

#### 3.3.5. Long-Run Estimation Results

After the cointegration test, the next suggested step is to estimate the long-run association between the study variables. For this purpose, this study relied on the recent estimation technique of MM-QR developed by Machado and Santos [41]. The benefit of this method is that it provides information on how the regressors affect the entire conditional distribution. FMOLS and DOLS methods are used as a robustness check, efficiently countering possible endogeneity issues.

#### 3.3.6. Panel Granger Causality Test

If government decision makers want to formulate policies and regulations with immediate effects, they must understand the factors of increased carbon emissions. Regression analyses can usually only yield contemporaneous correlations between different variables and, in order to analyze causal relationships between variables, this paper adopted the Dumitrescu–Hurlin [42] heterogeneous panel causality test method. This research approach gives two statistics, Zbar-stat and W-stat. When the value of Zbar-stat is greater than that of W-stat, the null hypothesis is rejected, and Granger causality exists. The formulation of the model can be depicted as below:

(7) $$z_{i,t}=\alpha_{i}+\sum_{j=1}^{p}\beta_{i}^{j}z_{i,t-j}+\sum_{j=1}^{p}\gamma_{i}^{j}T_{i,t-j}$$

## 4. Results and Discussion

Table 2 shows the descriptive statistical results for the above variables. In order to avoid the heteroscedastic problem, the above variables are processed by logarithmic transformation. Among all variables, the mean value of environmental regulations is the lowest, and there is a negative value of −0.335. Economic growth has the lowest standard deviation, which means that GDP is the least volatile series. The pairwise correlation indicates that environmental regulations and green innovation are negatively correlated to CO_2_ emissions, while capital formation, renewable energy, and economic growth positively correlate to CO_2_ emissions in G7 countries. It can be seen from Table 3 that the correlation coefficients between independent variables are all less than 0.8, which indicates that there is no serious multi-collinearity problem.

The test results in Table 4 indicate the presence of cross-section dependence between variables and accept the alternative hypothesis. The statistical results may be biased if the cross-section dependence is not controlled. To avoid the above situation, the cross-sectional dependency method of Pesaran [37] is used. Table 5 represents the results of slope heterogeneity test, which is acquired by using the Pesaran and Yamagata [29] test. The findings indicate the presence of slope heterogeneity in the panel of G7 countries.

In addition to considering cross-sectional dependence and heterogeneity, the stationarity of the series should be further tested. In order to avoid the phenomenon of spurious regression and ensure the validity of each variable parameter, the most commonly used unit root test method was used to test the stationarity of the series. In this paper, the CIPS and CADF methods of unit root Pesaran [30] are used to check whether there is a unit root in the sequence. The results of CIPS and CADF unit root tests show that the variables have mixed integration order. However, after taking the first difference, all variables are stationary. The results of the unit root test are shown in Table 6.

The results of the panel cointegration test are shown in Table 7, indicating the linear combinations between the variables have a stable equilibrium relationship. Thus, we can move forward to estimate the long-run relationship between study variables.

In this paper, the long-run relationship between the above variables is tested by MM-QR method, and the FMOLS and DOLS methods are used as a robustness check. The MM-QR provides an additional tool for estimating regression quantiles. Even if valid only for estimating the conditional mean, the method works equally well and still provides information about how the regressors affect the entire conditional distribution. 

As shown in Table 8, the MM-QR regression results show that the regression coefficient of capital formation to CO_2_ emissions is significantly negative at all quantiles. The regression coefficient value decreases from low to high quantiles. This implies that capital formation is negatively related to emissions in G7 countries. These findings can be justified on the grounds that capital formation in these countries is environmentally friendly, and these countries are investing in cleaner technologies to promote a low-carbon economy. Our findigs are similar to Ajide and Ibrahim [43], who found that capital investment can positively contribute to environmental quality in OECD countries. However, this diverges from the work of Mujtaba et al. [13], who argue that gross fixed capital formation hinders sustainable development.

In addition, the regression coefficients of environmental regulations for CO_2_ emissions are also significantly negative in each quantile, and the regression coefficients increase from low quantiles to high quantiles. This shows that implementing environmental regulations can effectively curb the increase in carbon emissions, and the correlation between the two is becoming stronger. This is consistent with the conclusion of the study of Pei et al. [44], who concluded that environmental regulations can regulate the emission behavior of enterprises, thereby directly reducing carbon emissions. In addition, environmental regulations can reduce carbon emissions by improving technical efficiency. Hassan et al. [45] stressed that the current tax management of environmental regulations does not effectively address the problem of carbon emissions. Therefore, government departments need to strengthen the stringency of environmental regulations and reduce carbon emission intensity by formulating realistic and effective environmental regulations.

Concerting the impact of green innovation on CO_2_ emissions, the coefficient values are significantly negative at all quantiles. This fully explains that the green innovation strategy of G7 countries plays an important role in reducing carbon emissions. This result is thus reasonable due to the fact that green innovation helps to produce modern and efficient technologies, which are capable of playing an important role in the preservation of the environment. In addition, these findings indicate that the G7 countries are on pace to reduce their CO_2_ emissions via the development of environmentally related technologies. Moreover, the existence of such technologies will assist to minimize the harms caused by economic advancement, and at a certain degree of progress, it will be possible to establish a negative connection between economic progress and CO_2_ emissions. Our results are consistent with the outcome of Ahmed et al. [22] and Hongqiao et al. [46], who also disclosed a mitigating effect of green innovation on environmental pollution.

The regression coefficient of CO_2_ emissions from renewable energy consumption is also significantly negative in each quantile. This phenomenon shows that the development and utilization of renewable energy has a positive effect on reducing carbon emissions. Compared with traditional energy, renewable energy resources have great potential, less environmental pollution, and can be used sustainably. They have the dual advantages of improving environmental quality and solving energy crises. However, in terms of long-term development, in order to effectively control carbon emissions in the long term, consumption of renewable energy is only one of the measures, which also needs to be considered together with technological development and other policies [47,48].

Eventually, the regression coefficient of economic growth on CO_2_ emissions is significantly positive through all quantiles. Clearly, the traditional business model achieves economic growth at the cost of energy consumption and environmental damage. The regression coefficient of GDP on carbon emissions has a downward trend from low quantile to high quantile, based on which it can be inferred that when GDP reaches a certain threshold, carbon emissions are likely to be reduced. This conclusion is supported by the opinions of Ahmad et al. [22] and Sharif et al. [49], who contend that the G7 energy mix is still largely dependent on fossil energy, even in recent years, and that the G7 countries have accomplished the majority of their growth by utilizing fossil energy that pollutes the environment. Therefore, economic development is now detrimental to environmental quality.

In summary, it can be seen that green innovation, renewable energy consumption, and environmental regulations have significant negative effects on carbon emissions in all quantiles. However, from low quantiles to high quantiles, the role of environmental regulations in reducing carbon emissions has gradually increased, and the role of other variables on carbon footprints has gradually weakened. Therefore, it also reflects the importance of environmental regulations in carbon reduction.

**Figure 1:**
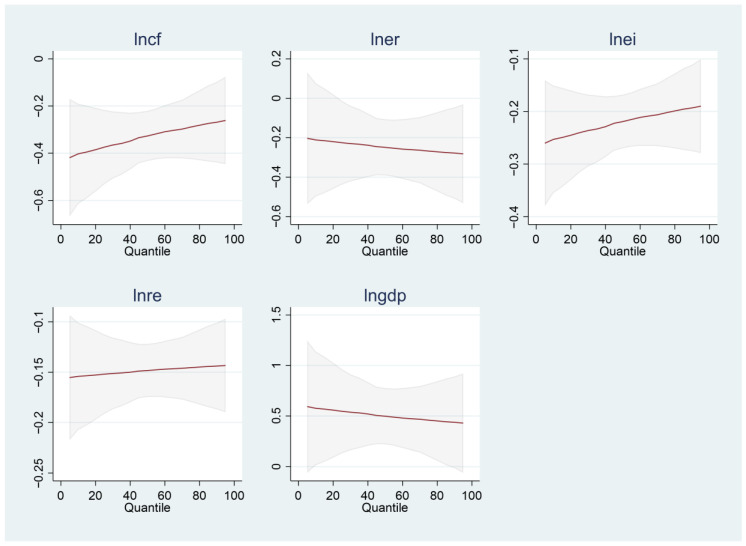
Quantile graphs.

Figure 1 shows the quantile map of each variable at each percentage level, describing the heterogeneous impact of capital formation, environmental regulations, green innovation, renewable energy consumption, and economic growth on carbon emissions. The effect of each variable and carbon emissions was conducted by three upper, middle, and lower quantiles.

Table 9 shows the FMOLS and DOLS test results. It can be seen from the results of the two research models that the statistical results of DOLS have a higher degree of correlation with carbon emissions than those of FMOLS. Environmental regulations, green innovation, and carbon emissions are all negatively correlated, which means that environmental regulations can help reduce carbon emissions, and the implementation of green innovation can also promote carbon emission capacity. From the perspective of renewable energy consumption, the consumption of renewable energy plays a significant role in reducing carbon emissions. In FMOLS and DOLS, the use of renewable energy reduced carbon emissions in G7 countries by 15% and 19%, respectively. The coefficient of GDP is positive in both models, which indicates that economic growth is accompanied by an increase in carbon emissions. Thus, the robustness test also validates the findings of MM-QR.

After the long-run estimation, it is imperative to understand the causal relationship between various variables and formulate development policies in line with the reality if they want environmental regulations to exert the expected effects. For this purpose, Dumitrescu–Hurlin panel Granger causality methods were used to examine the causal association between variables, and the output results are shown in Table 10. From the results in the table, we can notice that for G7 countries, there is unidirectional causality from capital formation to carbon dioxide emissions, and there is no feedback effect. Moreover, there is unidirectional causality from environmental regulation, green innovation, and renewable energy consumption to carbon dioxide emissions. This implies that any policy related to capital formation, green innovation, and environmental regulations can Granger cause CO_2_ emissions but not the other way round. The causal relationship between economic growth and carbon dioxide emissions is bidirectional, which means that any policy related to economic growth will lead to carbon dioxide emissions, and policies related to carbon dioxide emissions will also affect GDP.

## 5. Conclusions

This study is devoted to examining the impact of capital formation, environmental regulations, renewable energy consumption, and economic growth on CO_2_ emissions in G7 countries over the period between 1994 and 2019. In the process of empirical analysis, this study employed second-generation estimation methods for cross-sectional dependence, slope heterogeneity, CIPS and CADF unit root test, and Westerlund co-integration. In addition, the long-run relationship between the study variables was tested by MM-QR. In addition, the robustness test was used to estimate the long-run elasticities among the variables.

Through the above empirical analysis, the research conclusion can be drawn as follows. There is a significant negative relationship between capital formation and carbon emissions, that is, capital formation can reduce carbon dioxide emissions and improve environmental quality. Moreover, the results unfold that there is a significant negative relationship between environmental regulations, green innovation, and carbon emissions, that is, the implementation of green innovation can hamper carbon emission, and the enactment of environmental regulations can help reduce carbon emissions. Compared with the implementation of green innovation, the enactment of environmental regulations has a more obvious effect on reducing carbon dioxide emissions. Economic growth positively related to CO_2_ emissions in G7 countries. DH test shows that any policy related to carbon dioxide will affect GDP; conversely, any policy promoting economic development will also lead to carbon dioxide emissions, while policies related to capital formation, environmental regulations, and renewable energy Granger cause CO_2_ emission but not vice versa. 

Based on the results, this study proposed that blindly pursuing economic benefits can no longer meet the needs of countries to develop sustainable economies. At present, the ultimate goal of governments is to achieve economic growth and environmental improvement in parallel. Thus, policymakers can use environmental regulations as an important tool to protect the environment and ensure sustainable green growth. Therefore, governments of all countries should introduce relevant environmental laws and regulations according to local conditions, stimulate the willingness of enterprises to generate green innovation, attract enterprises to pay attention to investment in green technology innovation, and create a good institutional system for the sustainable development of domestic economies. In addition, environmental law enforcement supervision cannot be ignored. Through environmental supervision, enterprises should be timely constrained and guided in their bad economic behaviors, and a fair and transparent reward and punishment mechanism should be established. The effective implementation of regulatory policies cannot be separated from the cooperation of enterprises. The introduction of environmental laws and regulations drives enterprises to develop in the direction of environmental protection innovation. In the green innovation strategy, it is easier for enterprises to seize development opportunities and obtain investment opportunities. Promoting the application of green technology can bring long-term benefits to enterprises. Therefore, the development focus of an enterprise is to improve the ability of independent innovation in order to achieve three-dimensional energy saving and comprehensive carbon reduction. Enterprises must tap excellent scientific researchers, seek stable investment and high-quality innovation resources, make research and development match with practice, realize the transformation of technical theory into application, and create competitive advantages.

This study investigated the impact of capital formation, green innovation, and environmental regulation on CO_2_ emissions for a sample of G7 countries in the presence of some limited variables such as renewable energy consumption and economic growth. Future studies may consider other factors, such as human capital, political stability, financial risk, etc., for interesting findings. Additionally, the results of this study are important for G7 as well as other developed countries. Thus, future studies on these variables for countries such as developing and emerging groups, using different estimation methods may provide some new dimensions for improving environmental sustainability.

## Figures and Tables

**Table 1:** Variables description.

Variable	Abb.	Proxy	Data Source
Environmental quality	CO_2_	CO_2_ emission (tones per person)	[33]
Capital formation	CF	Gross capital formation (constant 2015 USD)	[34]
Environmental regulation	ER	Environmental taxes	[35]
Green innovation	GI	Patents on environmental technologies (% of total)	[36]
Renewable energy	RE	Renewables per capita (kWh—equivalent)	[33]
Economic growth	GDP	Per capita (constant 2010 USD)	[34]

**Table 2:** Descriptive statistics.

Variable	Obs	Mean	Std. Dev.	Min	Max
lnCO_2_	182	2.330	0.409	1.579	3.060
lnCF	182	27.201	0.777	25.881	29.096
lnER	182	0.595	0.432	−0.335	1.28
lnGI	182	2.251	0.287	1.627	2.754
lnRE	182	1.381	1.082	−1.215	3.595
lnGDP	182	10.624	0.131	10.306	10.931

**Table 3:** Pairwise correlations.

Variables	lnCO_2_	lnCF	lnER	lnGI	lnRE	lnGDP
lnCO_2_	1.000					
lnCF	0.414	1.000				
lnER	−0.817	−0.678	1.000			
lnGI	−0.137	0.077	−0.163	1.000		
lnRE	0.404	−0.133	−0.462	0.349	1.000	
lnGDP	0.434	0.592	−0.787	0.537	0.462	1.000

**Table 4:** Cross-sectional dependency test results.

Variable	CD-Test	*p*-Value	Mean Abs (ρ)
lnCO_2_	19.072 *	0.000	0.82
lnCF	7.942 *	0.000	0.51
lnER	8.231 *	0.000	0.50
lnGI	21.812 *	0.000	0.93
lnRE	9.413 *	0.000	0.71
lnGDP	18.327 *	0.000	0.78

Note. * <1%.

**Table 5:** Testing for slope heterogeneity.

Test	Value	*p*-Value
$\tilde{\Delta }$	10.702 *	0.000
${\tilde{\Delta }}_{adjusted}$	12.519 *	0.000

Note. * <1%.

**Table 6:** Panel unit root test.

Variable	CIPS		CADF	
	I	I (0)	I	I (0)
lnCO_2_	−2.124	−4.717 *	−2.155	−3.818 *
lnCF	−1.524	−3.442 *	−1.782	−3.201 *
lnER	−1.496	−4.382 *	−1.564	−2.573 **
lnGI	−3.233 *	−4.706 *	−3.359 *	−3.938 *
lnRE	−3.299 *	−5.604 *	−1.555	−4.094 *
lnGDP	−1.409 *	−3.768 *	−1.856	3.492 *

Note. * <1%, and ** <5% significance.

**Table 7:** Westerlund co-integration test results.

Statistic	Value	Z-Value	Robust *p*-Value
*G_t_*	−3.430 ***	−1.195	0.083
*G_a_*	−11.102 ***	2.342	0.075
*P_t_*	−9.107 **	−1.743	0.040
*P_a_*	−10.890 ***	1.405	0.072

Note: ** <5%, and *** <10% significance. Six-hundred replications utilized for bootstrapping.

**Table 8:** MM-QR regression results.

	Low			Medium			High		
	Q_0.10_	Q_0.20_	Q_0.30_	Q_0.40_	Q_0.50_	Q_0.60_	Q_0.70_	Q_0.80_	Q_0.90_
lnCF	−0.403 * [0.108]	−0.385 * [0.090]	−0.365 * [0.072]	−0.349 * [0.061]	−0.327 * [0.054]	−0.309 * [0.057]	−0.298 * [0.063]	−0.281 * [0.075]	−0.269 * [0.087]
lnER	−0.211 [0.146]	−0.220 *** [0.122]	−0.230 ** [0.097]	−0.238 * [0.082]	−0.249 * [0.072]	−0.258 * [0.077]	−0.263 * [0.085]	−0.271 * [0.102]	−0.278 ** [0.118]
lnGI	−0.253 * [0.052]	−0.245 * [0.043]	−0.236 * [0.035]	−0.229 * [0.029]	−0.219 * [0.026]	−0.211 * [0.027]	−0.206 * [0.030]	−0.199 * [0.036]	−0.193 * [0.042]
lnRE	−0.154 * [0.027]	−0.153 * [0.023]	−0.151 * [0.018]	−0.150 * [0.015]	−0.148 * [0.013]	−0.147 * [0.014]	−0.146 * [0.015]	−0.145 * [0.019]	−0.144 * [0.022]
lnGDP	0.576 ** [0.287]	0.558 ** [0.239]	0.537 ** [0.191]	0.520 * [0.160]	0.498 * [0.142]	0.479 * [0.151]	0.468 * [0.167]	0.451 ** [0.200]	0.438 *** [0.231]

Note: * <10%, ** <5%, and *** <10% significance.

**Table 9:** FMOLS and DOLS test results (Robustness check).

	FMOLS		DOLS	
Variable	Coefficient	Std. Error	Coefficient	Std. Error
lnCF	−0.336 *	0.033	−0.525 *	0.135
lnER	−0.278 *	0.042	−0.430 *	0.075
lnGI	−0.222 *	0.014	−0.334 *	0.035
lnRE	−0.151 *	0.007	−0.191 *	0.016
lnGDP	0.445 *	0.086	0.726 **	0.326

Note. * <1%, and ** <5% significance.

**Table 10:** Dumitrescu–Hurlin panel causality tests.

Null Hypothesis	W-Stat.	Zbar-Stat.	Prob.	Conclusion
lnCF ↮ lnCO_2_	5.553 *	8.517	0.000	lnCF →lnCO_2_
lnCO_2_ ↮ lnCF	3.483	1.312	0.189	
lnER ↮ lnCO_2_	6.570 *	8.614	0.000	lnER → lnCO_2_
lnCO_2_ ↮ lnER	2.979	0.782	0.434	
lnGI ↮ lnCO_2_	5.636 *	7.143	0.000	lnGI → lnCO_2_
lnCO_2_ ↮ lnGI	2.793	0.587	0.557	
lnRE ↮ lnCO_2_	3.338 *	3.524	0.000	lnRE → lnCO_2_
lnCO_2_ ↮ lnRE	3.639	1.477	0.139	
lnGDP ↮ lnCO_2_	7.386 *	9.898	0.000	lnGDP ↔ lnCO_2_
lnCO_2_ ↮ lnGDP	4.615 **	2.503	0.012	

Note. * <1%, and ** <5% significance.

## Data Availability

Data are available from the corresponding author upon reasonable request.
